# Durability of lopinavir/ritonavir dual-therapies in individuals with viral load <50 copies/mL in the observational setting

**DOI:** 10.7448/IAS.17.4.19799

**Published:** 2014-11-02

**Authors:** Nicola Gianotti, Alessandro Cozzi-Lepri, Andrea Antinori, Antonio Di Biagio, Maria Cristina Moioli, Silvia Nozza, Antonella Cingolani, Andrea De Luca, Giordano Madeddu, Stefano Bonora, Francesca Ceccherini-Silberstein, Antonella d'Arminio Monforte, Study on behalf of ICONA Foundation

**Affiliations:** 1 Infectious Diseases, San Raffaele Scientific Institute, Milano, Italy; 2 Infection and Population Health, UCL Medical School, London, UK; 3 Infectious Diseases, National Institute for Infectious Diseases “L. Spallanzani”, Roma, Italy; 4 Infectious Diseases Unit, University of Genoa, Genova, Italy; 5 Niguarda Ca’ Granda Hospital, Institute of infectious Diseases, Milano, Italy; 6 Infectious Diseases, Department of Public Health, Catholic University, Roma, Italy; 7 Infectious Diseases Unit, Siena University Hospital, Siena, Italy; 8 Department of Clinical and Experimental Medicine, University of Sassari, Sassari, Italy; 9 Infectious Diseases Unit, Department of Medical Sciences, University of Torino, Torino, Italy; 10 Department of Experimental Medicine, University of Rome Tor Vergata, Roma, Italy; 11 Clinic of Infectious and Tropical Diseases, San Paolo University Hospital Milan, Milano, Italy; 12 Health Sciences, University of Milan, Milano, Italy

## Abstract

**Introduction:**

We aimed at evaluating the efficacy and durability of a lopinavir/ritonavir-based dual regimen (LPV/r-DR) in virologically controlled HIV-infected individuals in current clinical practice.

**Methods:**

Patients who have initiated for the first time a LPV/r-DR with HIV-RNA<50 copies/mL were included in this observational study. The main endpoints were: time to virological rebound [VR=time of first of two consecutive viral loads (VL)>50 copies/mL] and time to experience either a single VL>200 copies/mL or discontinuation/intensification (= treatment failure, TF). Individuals’ follow-up accrued from the date of starting the LPV/r-DR to event or last available VL. Kaplan-Meier curves and Cox regression analysis were used. Covariates included in the multivariable analysis were gender, age, route of transmission, hepatitis co-infection, calendar year of starting the DR, nadir CD4+ count, VL at initiation of first cART, previous failures to protease inhibitors (PIs), time with undetectable VL before starting the DR and the type of DR [nucleoside reverse transcriptase (NRTI), non-NRTI (NNRTI), raltegravir or maraviroc, with NRTI as reference group]. Results are presented as median (Q1, Q3) or frequency (%) as appropriate.

**Results:**

108 individuals followed for 18 (7, 30) months were included; baseline (BL) characteristics are detailed in Table 1.

LPV/r was associated with a NRTI in 51, with a NNRTI in 10, with raltegravir in 29, and with maraviroc in 18 individuals. By 36 months from switching to the LPV/r-DR, the proportion of individuals with VR and TF was 10% (95% CI 3–17%) and 36% (95% CI 22–50%), respectively. We did not find any factor independently associated with the risk of VR. Older age (ARH=0.49 (95% CI 0.30–0.78) per 10 years older; p=0.003) was found to be protective from TF. Mean (SE) CD4+ cells/µL increase from BL to month 36 resulted significant: 195 (40.1) cells/µL (p=0.0028). We did not observe significant changes in AST, ALT, eGFR (MDRD formula), triglycerides and both total and HDL-cholesterol.

**Conclusions:**

A LPV/r-DR can be considered a valuable option in patients with HIV-RNA<50 copies/mL and ongoing toxicity from the third drug of the regimen, although up to 17% of patients showed viral rebound by 3 years. Older patients are at lower risk of failure with this strategy, but larger sample size is needed to identify who might benefit from this strategy instead of others.

**Table 1:** Baseline characteristics

Characteristic	NRTI (N=51)	NNRTI (N=10)	InSTI (N=29)	CCR5 antagonist (N=18)	Total (N=108)
Gender, n (%)					
Female	17 (33.3%)	3 (30.0%)	10 (34.5%)	1 (5.6%)	31 (28.7%)
Mode of HIV Transmission, n (%)					
IDU	15 (29.4%)	5 (50%)	11 (37.9%)	0	31 (28.7%)
Homosexual contacts	6 (11%)	1 (10%)	4 (13.8%)	14 (77.8%)	25 (23.1%)
Heterosexual contacts	17 (33.3%)	3 (30%)	13 (44.8%)	3 (16.7%)	36 (33.3%)
Other/Unknown	13 (25.5%)	1 (10%)	1 (3.4%)	1 (5.6%)	16 (17.8%)
Hepatitis co-infection (HCVAb+ or HBsAg+), n (%)					
yes	158 (29.4%)	3 (30%)	10 (34.5%)	0	28 (25.9%)
not tested	16 (31.4)	2 (20%)	13 (44.8%)	10 (55.6%)	41 (38%)
Calendar year of starting dual, median (Q1, Q3)	2011 (2008, 2012)	2011 (2004, 2011)	2012 (2010, 2012)	2012 (2011, 2012)	2011 (2010, 2012)
Age, years, median (Q1, Q3)	47 (40, 56)	42 (39, 48)	49 (46, 53)	44 (38, 50)	47 (41, 53)
CD4+ count at starting dual, cells/µ/L, median (Q1, Q3)	420 (294, 650)	712 (306, 884)	455 (282, 678)	610 (530, 640)	486 (305, 701)
ALT at starting dual, UI/L, median (Q1, Q3)	23 (17, 39)	43 (29, 56)	30 (18, 45)	18 (12, 21)	24 (17, 39)
AST at starting dual, UI/L, median (Q1, Q3)	23 (15, 43)	41 (28, 83)	28 (21, 53)	23 (19, 29)	27 (19, 46)
VL at initiation of first ART, n (%)					
100,000 copies/mL	11 (21.6%)	4 (40%)	7 (24.1%)	3 (16.7%)	25 (23.1%)
>100,000 copies/mL	6 (11.8%)	4 (40%)	2 (6.9%)	3 (16.7%)	15 (13.9%)
Unknown	34 (66.7%)	2 (20%)	20 (69%)	12 (66.7%)	68 (63%)
Follow-up for composite outcome (VL200 copies/mL or stop/intensification), months, median (Q1, Q3	18 (5, 30)	25 (9, 28)	11 (7, 33)	22 (10, 25)	18 (7, 30)
Other drug, n (%)					
Lamivudine	42 (82.4%)	0	0	0	42 (38.9%)
Emtricitabine	3 (5.9%)	0	0	0	3 (2.8%)
Tenofovir	6 (11.8%)	0	0	0	6 (5.6%)
Efavirenz	0	3 (30)	0	0	3 (2.8%)
Nevirapine	0	6 (60%)	0	0	6 (5.6%)
Etravirine	0	1 (10%)	0	0	1 (0.9%)
Raltegravir	0	0	29 (100%)	0	29 (26.9%)
Maraviroc	0	0	0	18 (100%)	18 (16.7%)
Previously virologically failed a PI, n (%)	6 (11.8%)	2 (20%)	1 (3.4%)	0	9 (8.3%)
Time with VL≤50 before switch to dual, months, median (Q1, Q3)	7 (1, 50)	38 (14, 60)	5 (2, 14)	7 (3, 19)	7 (2, 26)
CD4+ nadir, cells/µ/L, median (Q1, Q3)	302 (162, 527)	216 (92, 405)	356 (238, 642)	472 (348, 603)	347 (189, 544)
CD4+ at time of starting ART, n (%)					
200 cells/µ/L	14 (27.5%)	5 (50%)	7 (24.1%)	2 (11.1%)	28 (25.9%)
≥200 cells/µ/L	37 (72.5%)	5 (50%)	22 (75.9%)	16 (88.9%)	80 (74.1%)
eGFR at starting dual, mL/min/1.73m^2^, median (Q1, Q3)	82 (68, 109)	94 (59, 113)	78 (54, 102)	104 (93, 113)	88 (66, 110)
Total-cholesterol at starting dual, mg/dL, median (Q1, Q3)	200 (156, 232)	226 (170, 275)	183 (140, 231)	236 (188, 265)	200 (156, 236)
HDL-cholesterol at starting dual, mg/dL, median (Q1, Q3)	44 (37, 52)	54 (47, 80)	48 (37, 60)	44 (37, 50)	45 (37, 57)
Triglycerides at starting dual, mg/dL, median (Q1, Q3)	150 (101, 203)	154 (107, 232)	158 (113, 203)	186 (100, 206)	157 (108, 203)

